# “Late for testing, early for antiretroviral therapy, less likely to die”: results from a large HIV cohort study in China, 2006–2014

**DOI:** 10.1186/s12879-018-3158-x

**Published:** 2018-06-13

**Authors:** Houlin Tang, Yurong Mao, Weiming Tang, Jing Han, Juan Xu, Jian Li

**Affiliations:** 1 National Center for AIDS/STD Control and Prevention, Chinese Center for Disease Control and Prevention, Beijing, China; 2 University of North Carolina at Chapel Hill Project-China, Guangzhou, China; 3 School of Medicine, University of North Carolina at Chapel Hill, Chapel Hill, USA

**Keywords:** Late diagnosis, Early mortality, ART, China

## Abstract

**Background:**

Timely HIV testing and initiation of antiretroviral therapy are two major determinants of survival for HIV-infected individuals. Our study aimed to explore the trend of late HIV/AIDS diagnoses and to assess the factors associated with these late diagnoses in China between 2006 and 2014.

**Methods:**

We used data from the Chinese Comprehensive Response Information Management System of HIV/AIDS (CRIMS). All individuals who tested positive for HIV between 2006 and 2014 in China and were at least 15 years of age were included. A late diagnosis was defined as an instance in which an individual was diagnosed as having AIDS or WHO stage 3 or 4 HIV/AIDS, or had a CD4 cell count less than 200 cells/mm^3^ at the time of diagnosis.

**Results:**

Among the 528,234 individuals (≥15 years old) newly diagnosed with HIV between 2006 and 2014, 179,700 (34.0%) people were considered to have received late diagnoses. The late diagnosis rate decreased from 33.9% in 2006 to 29.7% in 2014 (*P* < 0.01). Late diagnoses were more likely to be found among those who were 45–54 years old (adjusted odds ratio [aOR]: 3.25, 95% confidence interval [CI]: 3.17–3.34) or 55+ years old (OR: 2.94, 95% CI: 2.86–3.02), male (aOR: 1.15, 95% CI: 1.13,1.17), employed as a farmer or rural laborer (aOR: 1.13, 95% CI: 1.11–1.14), infected through blood or plasma transfusion (aOR: 4.18, 95% CI: 4.02, 4.35), diagnosed at hospitals (OR: 1.17, 95% CI: 1.15, 1.19), of Han ethnicity (aOR: 1.30, 95% CI: 1.28, 1.32), and married (OR: 1.12, 95% CI: 1.11,1.13). Of those people living with HIV (PLHIV) who received late diagnoses, 7.4%(8637) and 46.1%(28,462) ultimately died with or without receiving antiretroviral therapy within a year of diagnosis, respectively.

**Conclusion:**

A large proportion of individuals with HIV/AIDS receive late diagnoses, and this proportion has witnessed a slight decline in recent years. Expanded testing is needed to increase early HIV diagnosis and antiretroviral therapy should be recommended to all diagnosed individuals as early as possible to reduce AIDS-related death.

## Background

The introduction and expansion of highly active antiretroviral therapy (HAART) has proven to be one of the most remarkable public health measures in reducing the morbidity and mortality caused by the Human Immunodeficiency Virus (HIV) [1–4]. UNAIDS sets an ambitious treatment target to help end the AIDS epidemic: By 2020, 90% of all people living with HIV know their HIV status, 90% of people with diagnosed HIV receive antiretroviral therapy (ART) and 90% of all people on HIV treatment achieve viral suppression [5]. However, delayed testing as well as stigma and discrimination against infected individuals places them at risk of receiving delayed antiretroviral therapy [6].

Early detection of HIV infection is a critical factor in controlling the spread of HIV [7]. Timely ART is associated with a better prognosis among HIV-infected individuals and lower rates of disease progression [8]. Prompt linkages with health care systems at the start of ART yields the maximum benefits of HIV medical treatment for people living with HIV (PLHIV) [9] while decreasing the sexual transmission rate of HIV [10–13]. A late diagnosis is detrimental to both the individual infected with HIV and the community for two reasons. First, in the absence of timely initiation of ART, the majority of patients will suffer the deleterious effects of HIV infection due to the gradual failure of the immune system, ranging from infection by opportunistic diseases to even death. Second, the propensity for HIV transmission by untested and hence unaware PLHIV is greater [14, 15] than for those made aware of their HIV status.

Developed countries such as the USA, Canada, and the European Union have been successful in addressing some of the key operational factors hindering the effective management of diseases affecting PLHIV [16–21]. However, it is estimated that 30% of PLHIV in the European Union [9] remain undiagnosed, while evidence from the United States suggests that 25% of the undiagnosed population is responsible for the transmission of new HIV infections in 54% of cases [7, 22].

The proportion of those who received late diagnoses in industrialized countries is most often reported at between 25 and 45% of all newly HIV-diagnosed cases [23]. Though the largest proportion of undiagnosed PLHIV resides in developing countries, little research describing their characteristics has been conducted in these settings. In China, HIV voluntary counseling and testing (VCT) is free and can be widely accessed in urban areas. However, this free testing strategy has not been able to prevent a large proportion of cases of late HIV diagnosis for several reasons [24]. Incomplete HIV screening among STD patients in China has been cited as one important cause of missed opportunities for HIV testing [25].

Our study aimed to identify characteristics and trends of cases of late HIV/AIDS diagnosis, and to assess the factors associated with late diagnosis, early mortality, and ART initiation in China between 2006 and 2014.

## Methods

### Data sources and definitions

In China, all newly diagnosed HIV/AIDS cases and all recipients of free ART are registered into the Chinese Comprehensive Response Information Management System of HIV/AIDS (CRIMS). CRIMS is managed by the National Centre for AIDS/STD Control and Prevention (NCAIDS) of the Chinese Centre for Disease Control and Prevention (China CDC) and includes the National Case Reporting Database (NCRD) and the National Free Antiretroviral Therapy Database (NFATD). The NCRD collects data on newly diagnosed HIV/AIDS cases, including demographic characteristics, route of infection, site of HIV diagnosis or blood sample source, date of diagnosis, laboratory test results with corresponding test date, and date of death. These cases are followed up every 6 months. During each follow-up, CD4 cell count, counseling and behavior intervention, and other referral services are performed. The NCRD also collects additional data during each follow-up visit, including changes in demographic information, disease stage, CD4 cell count, information on behavioral determinants, and details of ART. The NFATD includes all cases that meet the national treatment criteria and have received free treatment. In China, the national criteria for treatment is a CD4 cell count of less than 350 cells/mm^3^ (as of 2009) and later, of less than 500 cells/mm^3^ (as of 2014), or being reported as having AIDS, or having WHO stage 3 or 4 HIV/AIDS and having been referred for ART [26–29]. Information on the individual’s drug regiment during each follow-up visit is recorded and then uploaded to the CRIMS.

A detailed description of the NCRD and NFATD has been published elsewhere [26], including a discussion of quality control of lab work [28]. There have been several important ART studies based on the data from these two systems in recent years [27–29]. In this study, we updated the results in continuation with earlier findings from the cohort study [27]. We included all newly diagnosed HIV/AIDS cases between 1 January 2006 and 31 December 2014 in mainland China involving individuals aged 15 years and over and excluded individuals who could not be located by local CDCs during follow-up visits.

There were several explanations for the term “late diagnosis” [30–32]. In this study, we classified a case as a “late diagnosis” if an individual was reported as 1) having been diagnosed with AIDS or 2) having been diagnosed with WHO stage 3 or 4 HIV/AIDS, or 3) having an initial CD4 cell count of less than 200/mm^3^ within one year after the date of HIV diagnosis. Early mortality was defined as death from AIDS-related causes within one year of an HIV/AIDS diagnosis. Cases without a death certificate or without being lost to follow-up as of 31 December 2014, were assumed to be alive. Cases who died of overdose drug use or suicide were excluded.

We defined preventable early mortality as the estimated number of AIDS-related deaths within one year of diagnosis attributed to late diagnosis. The number of preventable early mortality equals to the number of observed AIDS-related deaths within one year of diagnosis minus the estimated number of AIDS-related deaths within one year of diagnosis according to the early mortality rate of those who received timely HIV diagnoses.

### Statistical analysis

We used SAS software (version 9.4; SAS Institute; Cary, NC) for data analysis and MapInfo Professional software (version 15.0; Pitney Bowes; USA) for maps creation. Descriptive analysis was done for late diagnoses across all cases, including gender, age at HIV diagnosis, marital status, education level, route of transmission, ethnic group, and screening sites. Screening sites were categorized into three groups: VCT sites, hospitals, and detention centers. Transmission routes were classified into five groups: heterosexual, homosexual, injection drug use, sexual contact and injection drug use, and former paid blood/plasma donation. Odds ratio (OR) was used to assess the factors associated with late diagnosis by means of univariate and multivariate logistic regression models. Variables for which *P* values were less than 0.05 were considered to be statistically significant.

## Results

**Fig. 1:**
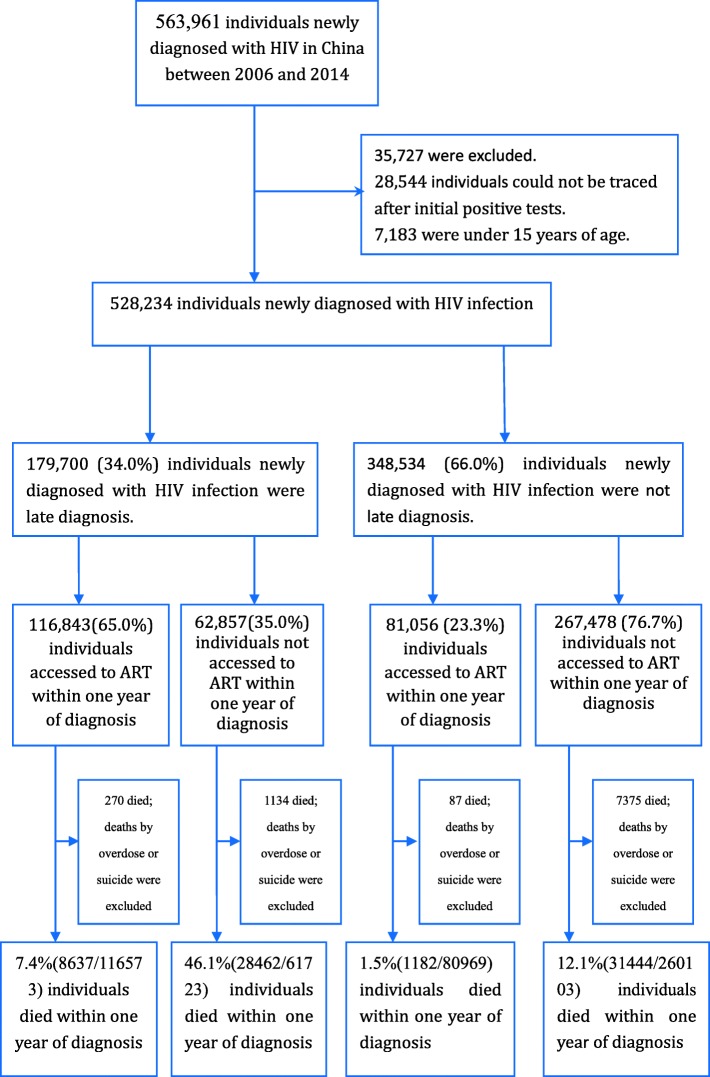
Study profile

A total of 563,961 individuals were newly diagnosed with HIV infection in mainland China during the period between 1 January 2006 and 31 December 2014. A total of 35,727 subjects were excluded from this analysis, as 28,544 cases could not be traced after the initial positive tests and 7183 cases concerned individuals were under 15 years of age (Fig. 1). Among the remaining 528,234 individuals, the majority were male (72.7%), married (50.2%), had attained a middle school level education or higher (60.8%), heterosexual (63.2%), and belonged to the Han ethnic group (74.6%). Most were diagnosed a t hospitals (44.7%) and VCT sites (28.4%) (Table 1).

**Table 1:** Demographic Characteristics and factors associated with late diagnosis in China between 2006 and 2014 (*N* = 528,234)

Categories	Newly diagnosed (age > 14 years old)	n (%) of Late diagnosis	Unadjusted OR (95%CI)	Adjusted OR (95%CI)
Total	528,234	179,700(34.0)	_	_
Year of HIV diagnosis				
2006	26,027	8823(33.9)	Ref.	Ref.
2007	32,538	11,427(35.1)	1.06(1.02–1.09)	1.04(1.00–1.08)
2008	40,690	13,734(33.8)	0.99(0.96–1.03)	0.93(0.89–0.96)
2009	48,163	17,073(35.4)	1.07(1.04–1.11)	0.99(0.95–1.02)
2010	53,375	19,700(36.9)	1.14(1.11–1.18)	0.99(0.96–1.03)
2011	67,256	23,944(35.6)	1.08(1.05–1.11)	0.89(0.86–0.92)
2012	76,716	26,598(34.7)	1.04(1.01–1.07)	0.84(0.81–0.87)
2013	85,518	29,291(34.3)	1.02(0.99–1.05)	0.82(0.79–0.85)
2014	97,951	29,110(29.7)	0.83(0.80–0.85)	0.65(0.63–0.68)
Sex				
Female	144,013	49,803(34.6)	Ref.	Ref.
Male	384,221	129,897(33.8)	0.97(0.95–0.98)	1.15(1.13–1.17)
Age groups(years)				
15–24	74,796	12,174(16.3)	Ref.	Ref.
25–34	163,089	44,161(27.1)	1.91(1.87–1.95)	187(1.82–1.91)
35–44	132,507	51,750(39.1)	3.30(3.22–3.37)	2.78(2.72–2.85)
45–54	70,158	32,262(46.0)	4.38(4.27–4.49)	3.25(3.17–3.34)
55+	87,684	39,353(44.9)	4.19(4.09–4.29)	2.94(2.86–3.02)
Marital status				
Single, divorced, or widowed	256,895	74,319(28.9)	Ref.	Ref.
Married or lives with partner	264,977	104,087(39.3)	1.59(1.57–1.61)	1.12(1.11–1.13)
Education				
Middle school or more	331,210	111,091(33.5)	Ref.	Ref.
Primary school or less	191,811	67,212(35.0)	1.07(1.06–1.08)	0.87(0.86–0.89)
Occupation				
Other	278,138	84,141(30.3)	Ref.	Ref.
Farmer or rural laborer	250,096	95,559(38.2)	1.43(1.41–1.44)	1.13(1.11–1.14)
Ethnic group				
Other	134,406	35,356(26.3)	Ref.	Ref.
Han	393,828	144,344(36.7)	1.62(1.60–1.64)	1.30(1.28–1.32)
Route of HIV infection				
Heterosexual	333,753	126,658(37.9)	Ref.	Ref.
Homosexual	85,252	20,891(24.5)	0.53(0.52–0.54)	0.71(0.70–0.73)
Injection drug use	73,834	14,111(19.1)	0.39(0.38–0.39)	0.63(0.61–0.64)
Sexual contact and injection drug use	5257	1147(21.8)	0.46(0.43–0.49)	0.71(0.66–0.76)
Blood or plasma transfusion	16,767	12,910(77.0)	5.47(5.28–5.68)	4.18(4.02–4.35)
Sites of diagnosis				
VCT centers	150,157	54,260(36.1)	Ref.	Ref.
Hospitals	236,047	97,220(41.2)	1.24(1.22–1.25)	1.17(1.15–1.19)
Detention centers	43,231	5087(11.8)	0.24(0.23–0.24)	0.35(0.34–0.37)
others	98,799	23,133(23.4)	0.54(0.53–0.55)	0.59(0.58–0.61)
Migrant population^a^				
No	402,155	143,160(35.6)	Ref.	Ref.
Yes	126,079	36,540(29.0)	0.74(0.73–0.75)	0.94(0.93–0.96)

^a^Migrant population: people migrated from one county to another county and stay there for at least six months

**Fig. 2:**
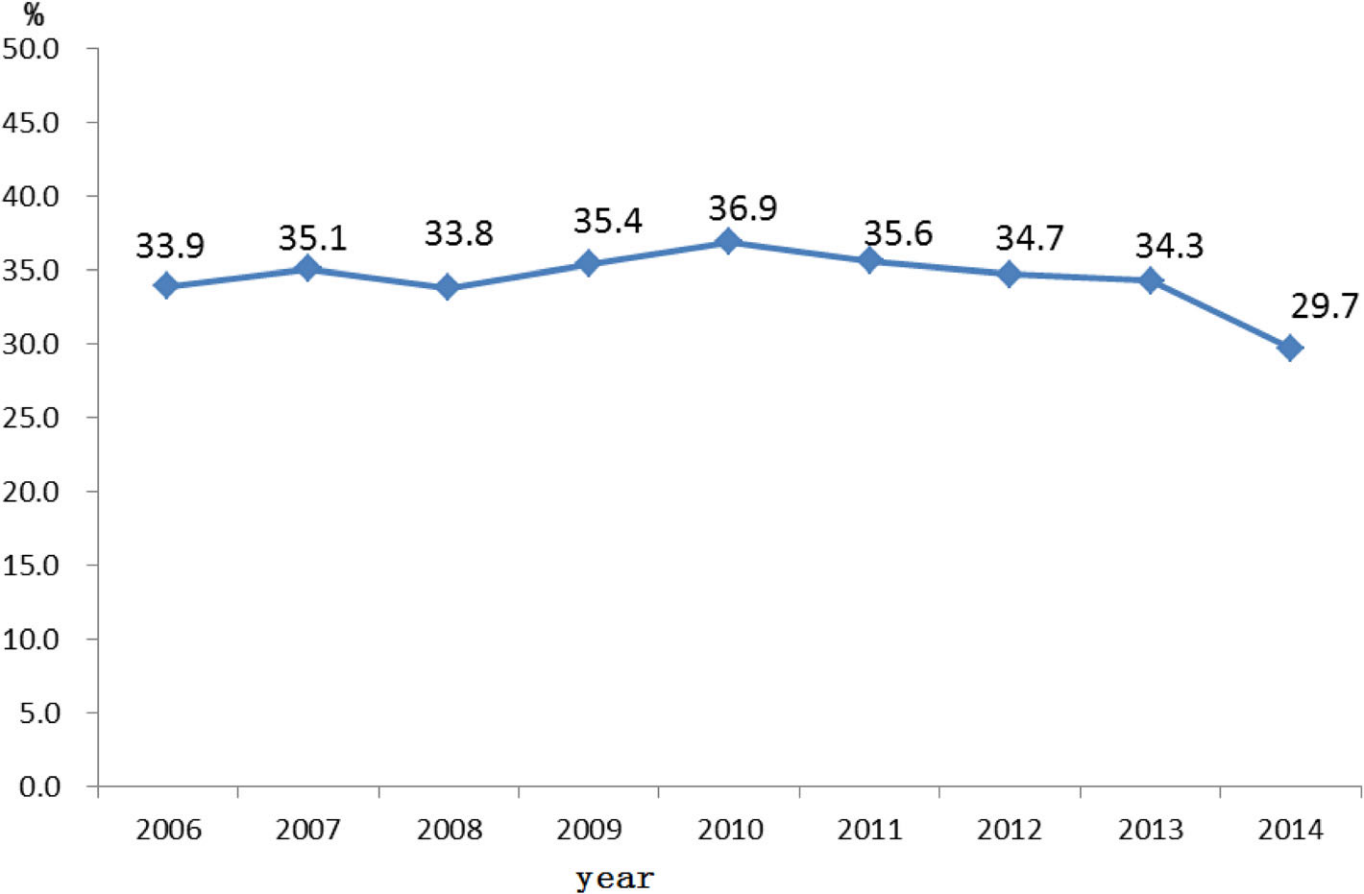
Proportions of individuals diagnosed late in different calendar years of diagnosis (X axis: calendar year of diagnosis; Y axis: proportion of late diagnosis)

**Fig. 3:**
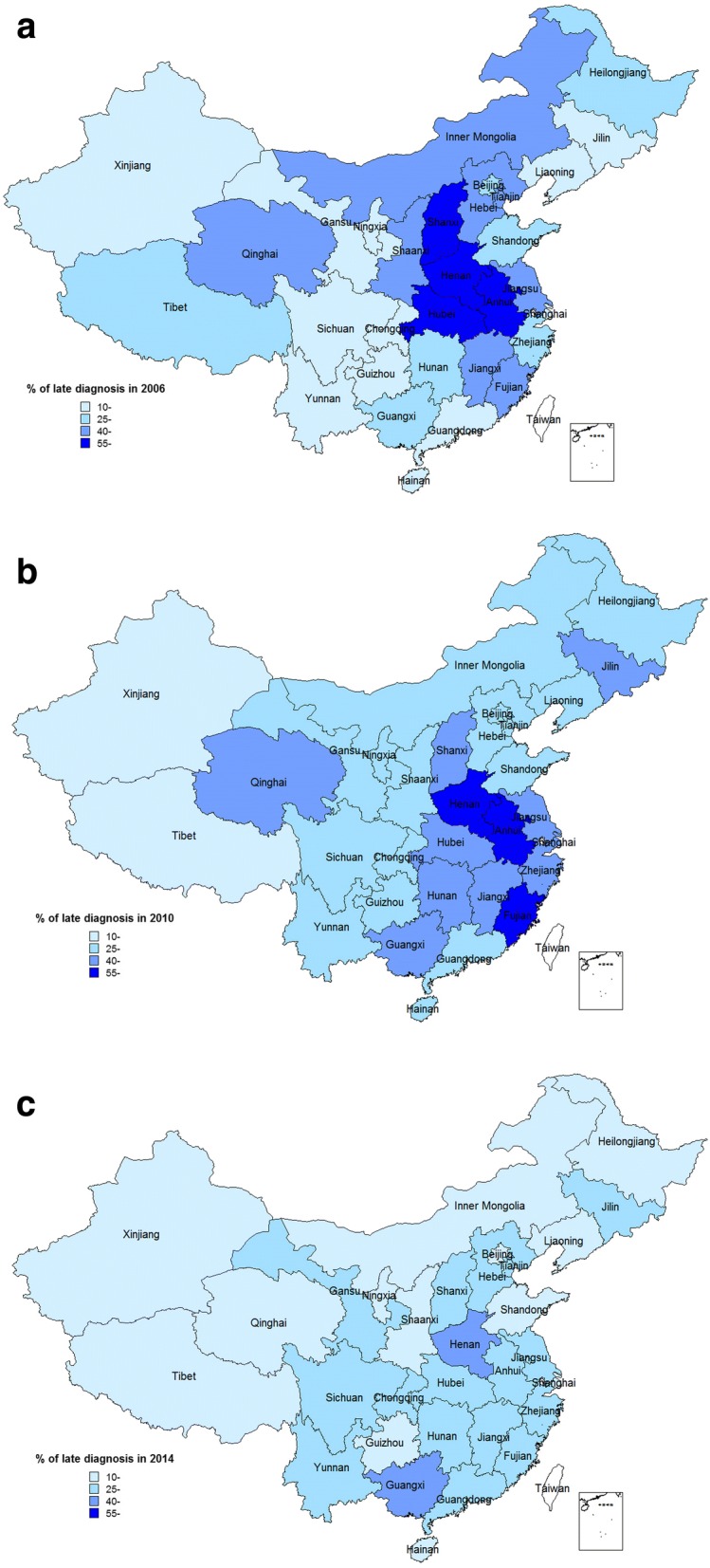
Proportions of individuals diagnosed late in different calendar years of diagnosis among 31 provinces in China (**a**: year of 2006, **b**: year of 2010, **c**: year of 2014)

In this cohort, 179,700 (34.0%) individuals newly diagnosed with HIV infection were classified as “late diagnosis” cases in accordance with the definition. The late diagnosis rate decreased from 33.9% in 2006 to 29.7% in 2014 (*P* < 0.01) (Fig. 2). The proportion of cases of late diagnosis were more than 55 % among four provinces in 2006, three provinces in 2010 and none in 2014 (Fig. 3).

The proportion of cases of late diagnosis was greater among those who were over 45 years old, married, farmers or rural laborers, and of Han ethnicity, infected through heterosexual transmission or former blood/plasma transfusion, diagnosed at hospitals, (Table 1). In addition, our results indicated that cases of late diagnosis increased with an increase in age in all calendar years during the study period.

As per our results, factors independently associated with an increased likelihood of late diagnosis were being male (aOR: 1.15, 95% CI: 1.13–1.17), being in an older age group (aOR: 3.25, 95% CI: 3.17–3.34 for 45–54 years old; aOR: 2.94, 95% CI: 2.86–3.02 for 55+ years old), having acquired HIV infection through former blood or plasma transfusion (aOR: 4.18, 95% CI: 4.02–4.35), being of Han ethnicity (aOR: 1.30, 95% CI: 1.28–1.32), being married or living with a partner (aOR: 1.15, 95% CI: 1.13–1.17), being farmers (aOR: 1.13, 95% CI: 1.11–1.14), and being tested at hospitals (aOR: 1.17, 95% CI: 1.15–1.19).

Around 52.0% of newly diagnosed cases (274,552) had their CD4 cell count results available within one month of HIV diagnosis (Table 2). This proportion increased from 19.0% in 2006 to 65.8% in 2014. Furthermore, this proportion was the lowest (29.7%) among those who were infected through injection drug use. Those who were diagnosed at VCT clinics received a timely (within one month) CD4 cell count testing of 60.9%.

**Table 2:** Initial CD4 cell counts after diagnosed of HIV newly diagnosed individuals in China between 2006 and 2014 (*N* = 528,234)

Characteristic	Newly diagnosed (age > 14 years old)	Initial CD4 cell count within one month		Initial CD4 cell count within one year		Initial CD4 cell counts < 200 cell/ul within one year	
		n	%	n	%	n	%
Year of HIV diagnosis							
2006	26,027	4950	19.0	9129	35.1	3901	42.7
2007	32,538	8856	27.2	15,621	48.0	6282	40.2
2008	40,690	14,475	35.6	23,979	58.9	8491	35.4
2009	48,163	20,327	42.2	32,083	66.6	11,321	35.3
2010	53,375	25,110	47.0	40,073	75.1	13,511	33.7
2011	67,256	35,950	53.5	54,378	80.9	18,002	33.1
2012	76,716	45,550	59.4	66,064	86.1	20,444	30.9
2013	85,518	54,931	64.2	76,191	89.1	23,474	30.8
2014	97,951	64,403	65.8	86,482	88.3	25,669	29.7
Route of HIV infection							
Heterosexual	333,753	183,602	55.0	264,332	79.2	96,527	36.5
Homosexual	85,252	57,299	67.2	78,895	92.5	15,515	19.7
Injection drug use	73,834	21,958	29.7	40,713	55.1	9168	22.5
Sexual contact and injection drug use	5257	1760	33.5	3257	62.0	777	23.9
Blood or plasma transfusion	16,767	7006	41.8	11,833	70.6	7142	60.4
Sites of diagnosis							
VCT centers	150,157	91,425	60.9	124,603	83.0	39,141	31.4
Hospitals	236,047	118,702	50.3	176,834	74.9	73,114	41.3
Detention centers	43,231	12,397	28.7	23,395	54.1	3196	13.7
others	98,799	52,028	52.7	79,168	80.1	15,644	19.8
Total	528,234	274,552	52.0	404,000	76.5	131,095	32.4

Our results indicate that 76.5% (404,000) of newly HIV diagnosed individuals received testing for CD4 cell count within one year of HIV diagnosis (Table 2). Of these individuals, 32.4% (131,095) had a CD4 cell count less than 200 cells/mm^3^. The proportion with a CD4 cell count test administered within one year increased during the study period from 35.1% in 2006 to 88.3% in 2014, and the proportion of CD4 cell count less than 200 cells/mm^3^ decreased from 42.7% in 2006 to 29.7% in 2014. Those infected through former blood or plasma transfusion witnessed a high proportion of CD4 cell count less than 200 cells/mm^3^. The proportion of CD4 cell count less than 200 cells/mm^3^ increased with age, from 13.3% in 15–24 age group to 44.8% in 55+ age group.

### Early mortality and access to ART

Of the 179,700 individuals who received late diagnoses, excluding 1404 individuals who died of overdose drug use or suicide, the proportions receiving ART within one month of diagnosis and within one year of diagnosis were 30.1 and 65.4%, respectively. The percentage of the individuals who received late diagnoses, received ART, and died within one month of diagnosis was 0.51%. The percentage of the individuals who received late diagnoses, received ART, and died within one year of diagnosis was 7.4% (Table 3). 17.4% of individuals who received late diagnoses but did not receive ART died within one month of diagnosis, and 46.1% of those who received late diagnoses without ART died within one year of diagnosis. Upon further analysis, the early mortality was 20.8% (37,099/178,296) among those who received late diagnoses and 9.6% (32,626/341,072) among those with timely diagnoses (*P* < 0.01).

**Table 3:** Early mortality among late diagnosed individuals with or without ART, by month (*N* = 178,296)

Duration of time from diagnosis	Initial CD4 counts	ON ART			NO ART		
		N	No. of death^a^	% of death	N^a^	No. of death^a^	% of death
One month	0–49	33,897	325	0.96	15,344	2449	15.96
	50–99	19,220	63	0.33	7153	516	7.21
	100–199	37,747	44	0.12	13,934	330	2.37
	200–349	17,685	14	0.08	6718	81	1.21
	350–499	5135	3	0.06	3301	32	0.97
	500-	2041	0	0.00	1977	10	0.51
	No CD4 test	848	142	16.75	13,296	7316	55.02
	subtotal	116,573	591	0.51	61,723	10,734	17.39
Three months	0–49	33,897	1551	4.58	15,344	5705	37.18
	50–99	19,220	390	2.03	7153	1399	19.56
	100–199	37,747	283	0.75	13,934	1000	7.18
	200–349	17,685	61	0.34	6718	201	2.99
	350–499	5135	15	0.29	3301	77	2.33
	500-	2041	3	0.15	1977	34	1.72
	No CD4 test	848	398	46.93	13,296	9674	72.76
	subtotal	116,573	2701	2.32	61,723	18,090	29.31
Six months	0–49	33,897	2950	8.70	15,344	7963	51.90
	50–99	19,220	880	4.58	7153	2253	31.50
	100–199	37,747	678	1.80	13,934	1684	12.09
	200–349	17,685	147	0.83	6718	359	5.34
	350–499	5135	39	0.76	3301	157	4.76
	500-	2041	14	0.69	1977	70	3.54
	No CD4 test	848	560	66.04	13,296	10,838	81.51
	subtotal	116,573	5268	4.52	61,723	23,324	37.79
Twelve months	0–49	33,897	4521	13.34	15,344	9796	63.84
	50–99	19,220	1520	7.91	7153	3100	43.34
	100–199	37,747	1385	3.67	13,934	2654	19.05
	200–349	17,685	361	2.04	6718	628	9.35
	350–499	5135	107	2.08	3301	307	9.30
	500-	2041	53	2.60	1977	141	7.13
	No CD4 test	848	690	81.37	13,296	11,836	89.02
	subtotal	116,573	8637	7.41	61,723	28,462	46.11

^a^Those who died of overdose drug use or suicide were excluded

Among PLHIV who received late diagnoses, the proportion experiencing early mortality declined annually from 2006 to 2014 with an increase in the proportion receiving ART (Table 4). The early mortality rates for individuals who received late diagnoses were 29.5% in 2006 and 14.5% in 2014. At the same time, the treatment coverage rates for these individuals within one year of diagnosis were 39.5% in 2006 and 73.9% in 2014. The treatment coverage rate for those who reported having had sex with men was highest (82.8%) (*p* < 0.01), therefore, this group also experienced the lowest rate of early mortality (8.0%) (Table 4).

**Table 4:** Access to ART and early mortality among late diagnosis individuals within one year of diagnosis between 2006 and 2014 (N=178,296)

Characteristics	Late diagnosis (age>14 years old)*	N (% of access to ART within one month)	N (% of access to ART within one year)	N (% of died after receiving ART)	χ^2^	P-value ^b^
Year of HIV diagnosis					141.52	<0.01
2006	8675	1436(16.6)	3429(39.5)	267(7.8)		
2007	11252	2105(18.7)	5126(45.6)	341(6.7)		
2008	13535	3051(22.5)	7387(54.6)	568(7.7)		
2009	16874	3906(23.1)	9527(56.5)	621(6.5)		
2010	19532	4498(23.0)	12021(61.5)	679(5.6)		
2011	23741	6383(26.9)	16355(68.9)	1076(6.6)		
2012	26445	8524(32.2)	19038(72.0)	1495(7.9)		
2013	29192	11068(37.9)	22212(76.1)	1897(8.5)		
2014	29050	12668(43.6)	21478(73.9)	1693(7.9)		
Sex					137.54	<0.01
Female	49561	15132(30.5)	32806(66.2)	1959(6.0)		
Male	128735	38507(29.9)	83767(65.1)	6678(8.0)		
Age group (years)					871.48	<0.01
15-24	12082	3107(25.7)	8305(68.7)	317(3.8)		
25-34	43688	12146(27.8)	28734(65.8)	1436(5.0)		
35-44	51244	15499(30.2)	33684(65.7)	2430(7.2)		
45-54	32084	10846(33.8)	21947(68.4)	1832(8.3)		
55+	39198	12041(30.7)	23903(61.0)	2622(11.0)		
Marital status					11.08	<0.01
Single, divorced, or widowed	73626	20957(28.5)	48061(65.3)	3411(7.1)		
Married or lives with partner	103411	32483(31.4)	67970(65.7)	5177(7.6)		
Education					488.54	<0.01
Middle school or more	110338	34898(31.6)	77177(69.9)	4792(6.2)		
Primary school or less	66599	18585(27.9)	39012(58.6)	3826(9.8)		
Occupation					663.26	<0.01
Other	83510	25305(30.3)	57787(69.2)	3130(5.4)		
Farmer or rural laborer	94786	28334(29.9)	58786(62.0)	5507(9.4)		
Ethnic group					16.57	<0.01
Others	34969	9013(25.8)	19866(56.8)	1335(6.7)		
Han	143327	44626(31.1)	96707(67.5)	7302(7.6)		
Route of HIV infection					622.49	<0.01
Heterosexual	126054	40124(31.8)	83978(66.6)	6547(7.8)		
Homosexual	20832	6951(33.4)	17167(82.4)	646(3.8)		
Injection drug use	13531	1955(14.4)	5809(42.9)	372(6.4)		
Sexual contact and IDU	1102	245(22.2)	560(50.8)	31(5.5)		
Blood or plasma transfusion	12841	3722(29.0)	7457(58.1)	917(12.3)		
Sites of diagnosis					681.88	<0.01
VCT centers	53825	17809(33.1)	36452(67.7)	2346(6.4)		
Hospitals	96608	29659(30.7)	62791(65.0)	5683(9.1)		
Detention centers	4859	197(4.1)	1563(32.2)	62(4.0)		
others	23004	5974(26.0)	15767(68.5)	546(3.5)		
Migrant population ^a^					10.03	<0.01
No	142108	43658(30.7)	93717(65.9)	7056(7.5)		
Yes	36188	9981(27.6)	22856(63.2)	1581(6.9)		
Initial CD4+ cell count					10298.66	<0.01
0-49	49241	18191(36.9)	33897(68.8)	4521(13.3)		
50-99	26373	9778(37.1)	19220(72.9)	1520(7.9)		
100-199	51681	17268(33.4)	37747(73.0)	1385(3.7)		
200-349	24403	6167(25.3)	17685(72.5)	361(2.0)		
350-499	8436	1177(14.0)	5135(60.9)	107(2.1)		
500-	4018	476(11.8)	2041(50.8)	53(2.6)		
No CD4 records	14144	582(4.1)	848(6.0)	690(81.4)		
Total	178296	53639(30.1))	116573(65.4)	8637(7.4)		

^a^Those who died of overdose drug use or suicide were excluded

^b^The proportion of those who died after ART within one year is compared by different characteristics

### Estimation of preventable early mortality

Early mortality among those who received timely HIV diagnoses was 9.6%. Basing our assumptions of previous research [30–32], we presumed in our study that 1) early mortality would be reduced to 9.6% if there were no late diagnoses; 2) there would be no effect on deaths occurring more than a year after diagnosis; and 3) there would no other competing risks. Based on these conjectures, only 17,116 deaths would have been expected from 2006 to 2014 within a year of diagnosis if the diagnosis was timely. That is, 19,983 deaths would have been avoided during this period, compared to the 37,099 deaths actually observed among the group with late HIV diagnoses. Therefore, the current burden of early mortality would be reduced by about 53.9%.

## Discussion

It has been thirty years since HIV testing first became available [33]. Despite the decades of availability of testing, it was estimated that at least one-fourth of PLHIV worldwide were unaware of their infection [24, 34–39], and that this ratio could be even higher in China [24].Our results indicate that promoting timely diagnosis and treatment provided a survival advantage to PLHIV, and that almost 54% of deaths that occurred within one year of HIV diagnosis could thus be prevented. Our study expanded upon the existing literature [16, 32, 37, 40, 41] by evaluating late diagnoses among all HIV cases identified between 2006 and 2014 in China, by assessing and mapping the trend of late diagnosis over time and by evaluating factors associated with late diagnosis among HIV cases in China.

In our study, 34.0% of HIV cases were considered to have received late diagnoses between 2006 and 2014. The late diagnosis rate fluctuated from 2006 to 2014, but slightly decreased from 36.9% in 2010 to 29.7% in 2014 (*P* < 0.01). This declining could be due to the implementation of the provider-initiated HIV testing and counseling (PITC) strategy since 2009. After the initiating of PITC, the HIV testing was significantly scaled up in most of provinces in China [37, 38, 42]. The scaling up of HIV self-testing in China could be another reason for this phenomenon. For example, around 30% of MSM in China self-reported that they ever self-tested in 2016 [36].

The propensity to receive late HIV testing was greater for older people, those who had acquired HIV infection through former blood or plasma transfusion or heterosexual behaviors, individuals of Han ethnicity, married individuals, and farmers. These results are consistent with the findings of previous studies [31, 43], with the exception of some studies in the U.S. indicated that younger people was more likely to receive late testing [44, 45]. In recent years, spouses’ and regular partners’ testing was scaled up in China, because of a series of national and provincial laws, regulations, and policy initiatives.

The majority of late diagnoses occurred among those who were infected with HIV through heterosexual behaviors. It indicated that there might have been a prolonged period of transmission risk to their sexual partners. Strategies, promoting rapid HIV testing at primary level of health institutions, promoting HIV self-testing, and increasing collaboration with community based organizations (CBOs), should be further implemented. Also, the risk of late diagnosis was significantly higher for those who were infected through former blood or plasma transfusion. Illegal paid plasma donation was prominent in central China in the 1990s and resulted in the infection of the majority of donors with the HIV virus [26, 46, 47]. Most of these PLHIV did not seek testing due to societal discrimination and stigma against HIV. As a result, they were diagnosed with HIV only after they experienced symptoms. Most of these people are located in four provinces in central China, as shown in Fig. 3.

However, the proportion of late testing has decreased among men who have sex with men (MSM) and injection drug users in recent years. This finding may have been due to ongoing intervention programs that promoted testing in these high risk groups [48, 49], such as methadone maintenance treatment (MMT) for injection drug users and free HIV testing for MSM provided by CBOs and VCT clinics. Similar results have been found in other studies [45].

Individuals who present at an advanced stage of immunosuppression are at high risk of clinical events and death and are more likely to have a poor response when they start ART [50, 51]. The proportion of people receiving late diagnoses among newly diagnosed individuals remained at more than 30% between 2006 and 2013. However, this proportion has been in decline since 2010. In an era where effective and free testing and free treatment options are available in China, it is alarming that there is a substantial proportion of people were late diagnosed, and they are at higher risk of early death. A records-based retrospective cohort study in China found that the highest mortality rates for AIDS-related death and all-cause death were found in the first year of follow up after HIV diagnosis [52]. This phenomenon could be explained by the fact that about half of the participating cases had already progressed to AIDS before being identified, progression to AIDS was one of the strongest risk factors for AIDS-related death, with an aHR of 7.42 [52].

Individuals who present at an advanced stage of immunosuppression are at high risk of AIDS related diseases and death as well as more likely to have a poorer response to ART [36, 53]. Both HIV testing and ART have been free for PLHIV in China since 2003 [54]. In spite of this policy, our results indicate that as many as 30% of all newly diagnosed individuals received late diagnoses. Shortening the time from infection to measurement of CD4 cell counts could be an important window for early treatment. We infer that the time from infection to CD4 measurement is a useful indicator for monitoring delays in access to HIV medical care among newly diagnosed PLHIV [55]. In our analysis, 52.0% of newly diagnosed individuals had their CD4 cell counts available within one month of HIV diagnosis. This proportion increased from 19.0% in 2006 to 65.8% in 2014. It is very important for late diagnosed individuals to receive CD4 cell count testing immediately, so that they can receive referrals for timely ART and thus reduce their risk of death. In our study, this proportion of cases received ART within one month after the diagnoses increased from 16.6% in 2006 to 43.6% in 2014. The average proportion of having CD4 counts within one year was 76% from 2006 to 2014. The causes of not having CD4 counts testing within one year included death before having CD4 counts, lost to follow up, and inconvenience to access to CD4 count test. To shorten the time to CD4 cell count testing and to improve access to regular testing and treatment, efforts were taken in the past few years to dramatically improve referrals and integration of patient tracking between the health facilities responsible for patients follow up, CD4 cell count testing, and ART delivery [56].

Our analysis suggests that groups at high risk of late diagnosis should be targeted for appropriate public health intervention and encouraged to seek earlier treatment. Scaling up HIV testing is one of the important strategies in implementing this goal. This strategy may bring out a greater proportion of hidden individuals infected with HIV [57]. Another strategy would be to consider population-wide screening for HIV, such as for patients in hospitals. In the U.S., the CDC recommends routine HIV counseling and testing in healthcare settings for patients aged 13–64 years, unless the local HIV prevalence is known to be less than 0.1% [1].

There are some limitations to consider while interpreting our findings. Firstly, 2.3% (12,352/528234) individuals were lost to follow-up after HIV diagnoses, and then CD4 cell counts data or having WHO stage 3 or 4 of them were not available at diagnosis. Thus, it is possible that some individuals with AIDS may have been misclassified as having HIV. However, the resulting misclassification will be non-differential and might bias the estimates towards the null, because the proportion of individuals misclassified does not depend on the individual with respect to other variables on the analysis [58]. Secondly, our results couldn’t assess differences in mortality in late diagnosed individuals who access to ART based on ART regimen. It needs further research.

## Conclusion

Even with these limitations, our results still indicate that it is important to increase the accessibility of HIV testing services. Routine testing for most individuals has been recommended as a cost-effective strategy and efforts to promote such testing should be developed [40, 53].

## Abbreviations

ART: Antiretroviral Therapy
CBOs: Community Based Organizations
CDC: Centre for Disease Control and Prevention
CRIMS: Chinese Comprehensive Response Information Management System of HIV/AIDS
HAART: Highly active antiretroviral therapy
HIV: Human Immunodeficiency Virus
MMT: Methadone Maintenance Treatment
MSM: Men Who Have Sex with Men
NCAIDS: National Centre for AIDS/STD Control and Prevention
NCRD: National Case Reporting Database
NFATD: National Free Antiretroviral Therapy Database
OR: Odds Ratio
PITC: Provider-Initiated HIV Testing and Counseling
PLHIV: People Living with HIV
VCT: Voluntary Counseling and Testing
